# Practical Remarks on Blood-Letting

**Published:** 1866-09

**Authors:** D. B. Trimble

**Affiliations:** Chicago, Ill.


### CHICAGO MEDICAL EXAMINER.

N. S. DAVIS, M.D., Editor.

VOL. VII. SEPTEMBER, 1866. NO. 9.

### Original Contributions.

### ARTICLE XXX.

### PRACTICAL REMARKS ON BLOOD-LETTING.

[CONCLUDED.]

By D. B. TRIMBLE, M.D., Chicago, Ill.

In many of the diseases of the *urinary* and *genito-urinary* organs, blood-letting is required.

In several of the diseases of the *kidneys*, it is very necessary.

In *acute nephritis*, if the patient is of vigorous constitution, and the attack severe, free general and local bleeding is necessary; but in milder cases, and in pyelitis, local bleeding is generally sufficient. Prout and Wood recommend this course. But not only in simple nephritis, but in inflammation of the kidneys in a previously diseased or degenerated state, especially if in an hæmotrophied condition, should also be "promptly met at the very outset by active antiphlogistic treatment. A few cases will require general blood-letting, and almost all will be benefited by local depletion; such as by cupping or leeching over the loins, etc." [Prout.] When the inflamed degenerated kidney is in the anæmotrophied condition, the same author remarks that general depletion must be more cautiously used; and cups or leeches applied over the loins, or leeches to the perineum or anus, will often be of great service. Roberts, in his work on "Urinary and Renal Diseases," recommends venesection in those cases of renal calculi accompanied by a "highly sthenic condition;" and cupping or leeching the lumbar region in those cases, and also in active congestion of the kidneys; in passive congestion, when produced by a temporary cause, as pregnancy, etc.; in suppurative inflammation, and in pyelitis.

In *nephralgia*, if accompanied by tenderness of the loins, cupping or leeching that region will sometimes be attended by relief.

*Ischuria renalis*, or suppression of urine, is occasionally, though but seldom, an idiopathic affection. When idiopathic, or when symptomatic, if caused by acute nephritis, or vascular irritation of the kidneys, or is attendant upon acute inflammation of other organs, or idiopathic fevers, blood should be freely taken from the arm, and the loins cupped. If owing to calculi in the pelves of the kidneys, or in the ureters, the same remedies, with a view to relaxation, as well as to subdue inflammation, may be required. When dependent upon cerebral or spinal disorder, cups or leeches should be applied to the base of the cranium, or to the vertebræ. When occurring in the latter stages of albuminuria, or in disorganization of the kidneys, or in Asiatic cholera, it would be useless and improper.

Of *Diabetes*, I can say but little from personal experience, as I recollect having seen but one well-confirmed case, in a girl 8 years of age, and which proved fatal. ROBERTS makes no observations on depletion in this disease; but PROUT remarks: "In cases of recent occurrence, and of an acute character, there cannot be a doubt about the propriety, and even necessity of blood-letting; which may be repeated as often as the circumstances of the case may seem to require. In very protracted cases, however, occurring in old subjects, and, indeed, wherever the debility is excessive, this remedy can be seldom required; though even in such cases it has been shown that blood-letting can be borne much better than could be expected. In most cases, frequent local bleedings from the epigastric region have been found beneficial; particularly when an extraordinary sense of fulness, heat, or tenderness has been experienced about the region of the stomach." Professor WOOD says that one of the indications for treatment is, "to alter the condition of the blood itself, which is probably the direct source of much of the functional and organic derangement which marks the progress of the disorder." This condition of the blood, he believes to be caused "by the loss of its water and salts through the kidneys, as well as by the imperfect assimilation of the nutriment thrown into it, the blood becoming depraved, and having an excess, probably, of the fibrinous and coloring ingredients." "Bleeding is the best palliative for this state of the blood. It tends to restore the equilibrium of the constituents; for the place of the blood abstracted is supplied with water and salts in larger proportion than with the animalized products. Nor does it materially weaken the patient. The vigor of the appetite, and the nutritious character of the food used, serve to obviate this result. The consequences are great relief to the uncomfortable feelings of the patient, a diminution of any inflammatory symptoms that may exist, and a reduction in the quantity of the urine." He then gives the same precautions in regard to its use that PROUT does.

In *hydruria*, or *simple diuresis*, venesection is seldom required; though where there is pain in the back, cups or leeches applied to the loins will often afford relief. I have had under my care, for six months past, a gentleman, 78 years of age, affected with severe diuresis, but in whose urine I could detect no trace of albumen, though several times subjected to the proper tests. His general health is much improved, pain entirely relieved, and the discharge of urine diminished, though still too great. Though suffering at first, and for some weeks, severe pains in the loins, and spasmodic pains in the neck of the bladder, I did not resort to local depletion, his age and debility precluding it. Narcotics and warm emollient applications, with infusions of uva ursi, or buchu, gradually relieved these symptoms; and tonics, carb. ammonia, and a generous diet are restoring him.

*Bright's disease of the kidneys.*—This term includes several pathological states of the kidneys, as albuminuria, different inflammatory conditions, and different kinds of degeneration; but ROBERTS, one of the latest authorities on renal diseases, classifies them, from a clinical point of view, as "acute and chronic Bright's disease." "The former embraces a compact and uniformly recognized group, which formerly went under the name of inflammatory dropsy." The latter includes the protracted cases, which he divides into three types. In acute cases, if seen at the "time of invasion," he recommends that the loins should be cupped to the amount of 8 or 12 ounces, to be repeated if there is severe headache, coma, or convulsions. In very severe cases, of the sthenic type, and high fever, venesection is required. In the young, the robust, or the temperate, PROUT recommends both general and local depletion; but in the intemperate, the debilitated, or the aged, local depletion only; or, in a few cases, general bleeding, moderately. WOOD, also, gives similar advice. In the chronic form, cupping or leeching the loins is recommended.

*Bladder.*—In *acute cystitis*, venesection, and leeches applied to the pubic or perineal regions, are indicated. In chronic cystitis, or *cystorrhœa*, the application of leeches is generally all that is required for direct depletion. In *irritable bladder*, if the patient is robust, and there is a sthenic condition of system, moderate venesection, or cupping the loins, or leeching the perineum may be beneficial; but as the disease generally occurs in the aged, it is seldom proper to resort to these measures. In *spasm of the bladder*, or *retention of urine*, when produced by an inflammatory condition of the organ, general and local bleeding is called for; and in robust persons, for spasm, venesection may also be practised, with a view to its relaxing effect, when there are no symptoms of inflammation.

In *prostatitis*, the same measures are applicable.

In *orchitis*, if the patient is young and robust, venesection, and repeated leeching of the scrotum, are indicated. Since writing the above, I have had a case of acute inflammation of the testicle, in a married man, of about 35 years of age, of correct moral habits. The testicle and scrotum were considerably enlarged and inflamed, and the former painful. There was no

*apparent* cause for it. I gave him saline purgatives, a local application of liq. plumb. sub-acet., and directed him to live on a light farinaceous and vegetable diet, and rest in the recumbent position. In one week, the inflammation had disappeared. I had a case, a few years ago, in a colored man, of enormous enlargement of both testes, from simple inflammation, without any syphilitic taint. When I first saw him, he had been sick about two weeks, and the scrotum was in a state of ulceration. The entire anterior portion of the scrotum sloughed off, completely exposing the testicles. He recovered after a tedious illness. In *sarcocele*, when either simple or scrofulous, and in *circocele* and *varicocele*, leeching the scrotum, or scarifying the veins, will often afford relief. In *hæmatocele*, if produced by rupture of the spermatic vein, and if the patient is young and not prostrated, venesection should be resorted to, and leeches applied, in case of inflammation of the scrotum after an operation.

*Hysteritis* may appear in the non-gravid womb, or as a sequel to parturition. In either case, venesection and local bleeding are necessary, but generally more promptly and freely in the latter than in the former case, on account of its greater activity and danger. The local bleeding may be accomplished by the application of leeches to the perineal, iliac, or sacral regions. BURNS, RAMSBOTHAM, MEIGS, and other good authorities recommend this treatment, and my own experience confirms it. In the earlier stages of *scirrhus*, or *cancer of the uterus*, "taking blood from the loins by cupping, or from the pubis or groins by leeches, is of service" to allay the throbbing, heat, or pain.

In *amenorrhœa*, if produced by vascular irritation of the uterus, accompanied with general plethora, pain in the back or head, and fever, venesection should be resorted to; and in *dysmenorrhœa*, the same conditions being present, leeching the pubis or perineum. In *leucorrhœa*, where there is a plethoric condition of system, venesection or leeching will be beneficial.

In diseases of the serous membranes terminating in *dropsy*, blood-letting is sometimes of great benefit; is, indeed, sufficient of itself, sometimes, to effect a cure. When caused by suppressed perspiration; from cold; or by the suppression of other secretions, as the menstrual; or by the vascular fulness attendant npon pregnancy; or from febrile, or local inflammatory affections; and attended by fever, or a full and tense pulse in persons of good constitution, and not too far advanced in age, venesection may be practised with benefit, with the view to diminishing the irritation of the secreting membranes; and, by relieving the fulness of the bloodvessels, promoting absorption of the effused fluid. If the blood exhibits the characteristic marks of inflammation, the bleeding may be repeated, but not frequently. The great majority of cases of dropsy do not require, and will not bear this remedy; but, when applicable, it sometimes acts with very prompt and beneficial influence. Where the serous membrane of any portion of the system is inflamed, topical depletion may be resorted to, cups being preferable to leeches. Where there is a torpid condition of the brain in general dropsy, cups to the temples or nucha may relieve temporarily. In hydrothorax, in its various forms, whether the effusion be in the parenchyma of the lungs, the pleuræ, or pericardium, cupping over the affected region will, sometimes, alleviate the symptoms; and in ascites, or in encysted ovarian dropsy, cups to the abdominal or pelvic regions will have a similar effect.

In *rheumatism*, if *acute*, bleeding may generally be practised with benefit. Persons affected with this form of the disease are, mostly, those in the vigor of life, of robust constitutions, and bear depletion well. The disease is almost always accompanied by fever of an active grade; and the pulse is full, frequent, and tense. One or two free bleedings, however, are all that is usually required; nor can we expect to permanently reduce the pulse by this means, without employing it to a dangerous extent. Too frequent bleedings have been supposed, in some cases, to cause metastasis to internal organs, especially to the heart, thus producing dangerous, sometimes fatal results. If this translation should take place in the course of the disease, not from the effects of bleeding, then venesection should be used as far as the system will bear it, followed, if the heart is involved, by cups between the shoulders, or to the left thoracic region; if the brain, by leeches to the temples, nucha, or scalp. In sub-acute, or chronic rheumatism, bleeding is seldom requisite.

Dr. FULLER, (of London,) in his work on "Rheumatism," by his extreme caution, almost ignores blood-letting in this disease; though, in cases occurring in the young and robust, and for the first time, he admits that one small bleeding may be useful. Even in rheumatic endo-carditis, or peri-carditis, or where the brain, lungs, or pleuræ are affected, he is not much more favorable to it, although he admits that local bleeding, especially leeching, may sometimes have a beneficial effect. In opposing the practice of BOUILLAUD, that of large and frequently repeated bleedings, he errs on the other hand, though not to so great an extreme. In this assertion, I think the experience of those who have had to treat acute rheumatism frequently, will support me, as my own certainly does.

Bleeding has no direct influence in the cure of *gout*, and should only be resorted to when the disease attacks some vital organ, as the heart, stomach, lungs, etc., when prompt and efficient venesection is sometimes required to save life. Nor must we be always guided by the state of the pulse, for though when full and strong there can be little doubt of its propriety, yet, sometimes, the depression is so great that the pulse becomes feeble and contracted, and the strength reduced. In the latter event, the flow of blood will sometimes be accompanied by an increased volume and firmness of the pulse, and the urgent symptoms be alleviated. After general bleeding, if the symptoms are not conquered, topical depletion over the affected organ may be used with benefit.

In the treatment of *idiopathic* fevers, blood-letting is of great benefit, sometimes indispensable. Under this head, I shall only consider those fevers that are unaccompanied by eruptions, leaving the latter to be treated of under another caption. There is a fever which I have sometimes witnessed, brief, often ephemeral, in duration; occurring most frequently in children; easily relieved, and rapidly convalescing. It is the same, probably, as that described by Dr. Wood, under the name of *irritative fever.* If in an adult, and the fever should be very high, with a full, strong pulse, severe headache, and tendency to cerebral congestion, one full bleeding from the arm may be requisite, but is not often necessary. I have had a case, within the last two weeks, in a young man, where there was a full and frequent pulse, hot skin, and flushed face, but with little headache. An active saline cathartic, followed by a cooling diaphoretic, which produced free perspiration, relieved him, and in 48 hours he was convalescent. In children, where convulsions are induced, and a similar condition of system pertains, leeches to the temples may be proper; but many of those cases of convulsions are occasioned by nervous disturbance alone, and then this remedy is not indicated.

In *miasmatic fevers,* direct depletion is not very frequently required. The indications for its use are, when the system is in a sthenic condition; the pulse full and tense, in the hot stage; and there is danger of inflammation of some internal organ, as the brain, stomach, liver, or lungs; or where the vomiting is incessant, and cannot be otherwise controlled. In the cold stage, when apoplectic symptoms occur, it may also be necessary. These remarks apply to each variety, the intermittent, the remittent, or congestive types, but more especially to the remittent. "Topical bleeding is of very great service, and of very general application. There are but few cases probably in which it may not be beneficially applied." [Bartlett's "Fevers of the U.S." The author's views in relation to bleeding in fevers, sustain the position taken in this article.] Where there is a tendency to inflammation of any of the vital organs, leeches or cups should be applied in the vicinity of such organ.

*Yellow fever* is a disease of such rare occurrence in this latitude, that a knowledge of its treatment is not of such importance as that of fevers of a milder type; yet, it may occur to others, as it has to myself in a solitary instance. In that case, I was embarrassed in my diagnosis, until I had the assistance of an old physician of Philadelphia, who was familiar with yellow fever, and who pronounced it to be that affection. This being my limited experience, I prefer to give that of others in regard to its treatment. Prof. WOOD says:—"The question must be decided at an early period, whether it will be requisite to use the lancet." "It is generally considered a hazardous remedy, after the lapse of one or two days. Bleeding will not *cure* the disease, nor should it be used vaguely, with this view. The only legitimate indication for it is to diminish the danger of disorganization from the violence of inflammatory excitement. If carried too far, it may do immense harm, by increasing the prostration of the second stage. It should be resorted to only when the pulse is tense and full. When this is very frequent, or readily compressible, the lancet should not be employed." "In the great majority of cases, it will not be necessary to bleed at all." In the case under my care, bleeding was not resorted to. When the yellow fever prevailed in Philadelphia, in 1794, the most eminent physicians there, Drs. RUSH, PHYSIC, DEWEES, and others, bled freely. In the years 1828, '29, and '30, experiments were made on some of our national vessels in the West Indies and Gulf of Mexico, to test the comparative merits of the antiphlogistic and mercurial treatments, and the result was greatly in favor of the former, in which venesection was almost invariably practised. Yet, the opinion of eminent physicians of the present day, who have had much experience in the disease, is adverse to bleeding, except in certain cases, (as before specified,) and to a moderate extent.

Although in *typhoid fever*, I have never, to the best of my recollection, resorted to bleeding, and have had few fatal cases in a moderately large number of patients, yet I have no doubt that in a few cases the cure would be accelerated by general, and in more by local depletion. If, in the early stage of this disease, there is a full, strong pulse, and a determination of blood to any of the vital organs, one or two moderate bleedings from the arm might be resorted to with probable advantage, with the view to the prevention of local inflammation. This should be used with great caution, however, as the disease is generally very protracted, and the debility, ultimately, very great. If inflammation, or active congestion of any of the vital organs should occur in the course of the disease, cups or leeches applied to the vicinity will often have a beneficial effect. Some of the French physicians, as BOUILLAUD, CHOMEL, and LOUIS, and, also, but to a less extent, some in New England, recommend depletion, while others, probably a large majority, reject it altogether. With Professor BARTLETT, I think that, "for the present, our management of the disease must be eclectic and rational, not exclusive and specific."

As in yellow, so in *typhus fever*, I cannot speak from much experience. It is a disease rarely prevailing in the localities where I have formerly practised. In regard to direct depletion in this affection, there has been a diversity of opinion, but it is now generally regarded as but seldom necessary. Where there is severe headache in the stage of reaction, or a danger of local congestion, and the pulse is strong, the abstraction of a few ounces of blood by venesection will, generally, relieve the former symptom, and tend to obviate congestion. When there is congestion of the brain, or other vital organ, the application of cups or leeches will often be beneficial.

*Eruptive diseases.*—Under this head, I shall treat of diseases of the skin, and also some of the *eruptive fevers*, as variola, rubeola, scarlatina, and erysipelas.

In *variola*, I have never bled, nor have I ever regretted doing so, except in one instance, which terminated fatally in a few hours from my first visit. The patient, in this case, was a plethoric woman, about 40 years of age, and a stranger to me. I found her slightly delirious, with a flushed countenance, contracted pupils, and a full and frequent pulse. She had some headache, and severe pains in the lumbar region. She attributed her sufferings to the non-appearance of her catamenia, and as there was no small-pox prevailing in the neighborhood, and no eruption visible, I did not suspect that disease. I prescribed for her (about 1 o'clock P.M.) and requested to be informed of her condition in a few hours. At 5 o'clock P.M., I received a message that she was much relieved, and had taken a little nourishment; but at 8 o'clock P.M., I was hurriedly summoned to her bedside, and found her in a dying condition. She lived about half-an-hour after my arrival. I then perceived on her face a papulous eruption, not very thick, however; and after death a few spots on the neck and chest. As many of the neighbors were crowding into the room, I considered it my duty to let them know that I thought the disease was small-pox. This offended the husband, who doubted her having the disease, and I requested that her former physician (a gentleman of large experience and reputation) might see the body with me. We met the next night, and, on examination, found her covered from the head to the feet with the variolous eruption, which had commenced to appear before life was extinct, and continued to increase up to the hour of our examination. Like myself, he had no hesitation in pronouncing it confluent small-pox of the most virulent kind. I afterwards found that the patient had spent a portion of a day, about ten days previously, in an infected part of Camden, N.J. In this case, venesection would, probably, have temporarily relieved the congested condition of the brain, have eliminated the eruption, and might have prolonged life; but the result would have ultimately, no doubt, have been the same. This is the only adult case I have ever lost by small-pox, and only two children, in a pretty large experience with the disease, and, therefore, that *experience* supports me in the opinion that it is very seldom necessary. Professor WOOD says, that "bleeding should never be used in the hope of eradicating the fever, or diminishing the amount of the eruption. It has been well ascertained to have neither of these effects, at least with the slightest approach to certainty." "Sometimes, when the eruption seems to be kept back by powerful internal irritation," (as in the case related above,) "bleeding has the effect of hastening it." "I repeat, the bleeding should be employed only to avert threatened danger from some internal organ. If there should be evidences of inflammatory congestion of the brain, lungs, stomach, or other important organ, accompanied by a full and strong pulse, he recommends moderate venesection, followed by leeches or cups near the affected organ. According to my own observation, this condition of the vital organs does not often occur to such an extent as to require bleeding. GREGORY, in his lectures on eruptive fevers, delivered in St. Thomas' Hospital, London, says:—"If the pulse be sharp, or very full, if the headache be severe, with accompanying epistaxis, blood-letting is not only useful, but *absolutely indispensable;* for the eruptive process is often impeded by the quantity of blood in the body, and the violence of the arterial excitement." "Your object is to unload the lungs, the liver, or the brain." It may be that, in hospital practice, and in our more plethoric transatlantic brethren, this condition of the system more generally obtains; but in private practice in this country, bleeding, according to my observation, is but seldom required.

In *Rubeola*, bleeding, for the disease itself, I believe, is never necessary. Some of its complications, or sequelæ, as pneumonia, laryngitis, bronchitis, etc., may require topical, possibly general, bleeding. A well-managed and well-nursed case of measles is, however, seldom followed by these diseases. Convulsions sometimes occur in children, when, if from a congested condition of the brain, especially in the early stages, leeches should be applied to the temples.

In regard to *scarlatina*, Dr. GREGORY remarks:—"I wish to impress on you, strongly, that scarlet fever not only admits of blood-letting, but often imperatively requires it, and that on general bleeding alone the safety of the patient often depends." He then gives a case approaching coma, another where there was great gastric irritation and vomiting, etc., and says:—"While I thus advocate the necessity of blood-letting in some cases, I freely acknowledge that it is inapplicable in others." Different epidemics, also, are affected differently by it, some bearing it well, others not at all. Professor WOOD says, that "it should be used with much caution and reserve, and only when there is an obvious indication; as when symptoms of inflammation of one of the vital organs exist and threaten great danger." I can say with Dr. WOOD, that "I have seldom found it advisable to bleed in any case; and do not remember the instance in which it appeared to me that I had occasion to repent my abstinence." I believe that it is the generally acknowledged practice of American physicians to abstain from general bleeding in scarlatina, as a rule. Where convulsions occur from cerebral congestion, or where the inflammation of the throat is severe, and not of a low or asthenic grade, leeches to the temples or throat will often afford much relief. It should be remembered that leech bites bleed very freely in scarlet fever, owing to the excited state of the cutaneous circulation, and the number used should not be great. When pseudo-membranous laryngitis accompanies, or supervenes upon, scarlatina, leeches may also be applied to the throat, though not with much prospect of success. In some of the other sequelæ, as pneumonia, otitis, or other inflammations, leeching is indicated.

*Erysipelas.*—The remarks made respecting depletion, in the three preceding diseases, will apply, generally, to erysipelas. It is only where, with inflammation or active congestion, some vital organ is dangerously affected, that leeching is indicated. In the only fatal cases of erysipelas that I have seen, death has been caused by a translation of the disease to the brain, and where there is a danger of this occurring, local, in some cases general, bleeding is required. Since my residence in Chicago, I had a severe case of erysipelas, in a young woman of good constitution, in which, on the third day of the eruption, violent cephalalgia, with great restlessness, occurred, which did not yield to the remedies administered, and which I feared might terminate in the translation of the disease to the brain. At a subsequent visit, the headache having increased, and the pulse being full and firm, I opened a vein in the arm and took 12 or 14 ounces of blood, with great, and speedy, and permanent relief. The patient recovered well, under the use of ferruginous tonics. But, except in these complications, I do not consider blood-letting necessary or advisable generally, though in the young or middle-aged, if the constitution is robust, the pulse full and hard, and the inflammation severe, bleeding will probably abbreviate its duration. On this subject there has been much diversity of opinion, probably owing to the different views of the nature of the disease. Those who regard it as only an inflammatory disease, would probably advocate blood-letting; those, again, who view it as a disease of debility, will resort to tonics and stimulants; while those who believe it is chiefly owing to a deranged condition of the digestive organs, especially the liver, will resort to measures calculated to relieve this condition. In different localities, the disease may assume one of these conditions to a greater extent than the others, and the treatment should be modified accordingly. As it has appeared to me, I have generally thought it comprised the three conditions combined, *viz:*—hepatic and gastric derangement; and inflammation, with a tendency to an asthenic state of the system. If this view is correct, bleeding would generally be unnecessary and injurious.

I have a few words to say in regard to blood-letting in *diseases of the skin*, and then these articles, which have been more numerous and more extended than I at first designed, will be brought to a close. In private practice, (at least, such has been the case with me,) there is not a large field for experience in diseases of the skin. As a general rule, I have seldom resorted to blood-letting, especially venesection, in this class of diseases. My chief authority for the following observations, is the work, on diseases of the skin, of Dr. NELLIGAN, of Dublin.

In *urticaria*, sometimes, the febrile symptoms are very active, in which case GREGORY, as well as NELLIGAN, advises venesection. The former particularly, "strongly counsels" it. WOOD advises it, "if the disease is obstinate and the patient plethoric."

In *eczema rubrum*, if the patient is of full habit of body, and there is much fever and pain, venesection may be required in some cases, in the early stages of the disease; but more frequently the topical abstraction of blood, by leeches, from the vicinity of the affected parts is sufficient, and is attended with much benefit.

Herpes rarely requires depletion. In the phlyctenoid and zoster varieties, where the fever is active, and occurring in young persons of full habit, venesection, or the application of leeches over the affected part, may be of use. These measures should not be resorted to, until the eruption appears. Neither GREGORY or WOOD advise depletion.

In *pemphigus*, this remedy is rarely required; but should the accompanying fever "continue after the bullæ are fully devel. oped, or inflammatory symptoms then appear, a small abstraction of blood from the arm may be requisite." [NELLIGAN.] WOOD recommends, "in the severe forms, where the pulse is strong and the constitution vigorous," to take blood from the arm, and by leeches "near the severest inflammation."

*Acne simplex*, generally requires very little treatment; but when it becomes obstinate, and "returns in a very active form every spring, a general bleeding, in strong, young persons, sometimes prevents its outbreak." WOOD recommends the same treatment, with the addition of leeches near the affected part, where the inflammation is severe and the patient vigorous, or plethoric.

*Impetigo*, occurring in young persons of robust constitution, sometimes requires local, even, in some cases, general bleeding. [NELLIGAN, WOOD.]

In *lichen agrius*, unless in "strong, healthy young persons, residing in the country," general bleeding is not required, "the local abstraction of blood, by means of leeches applied in the neighborhood of the eruption, being, in most cases, sufficient. Even in the chronic stages of the disease, this form of local bleeding is, in general, attended with the best results, as it relieves the congested state of the capillary circulation which is present." [NELLIGAN, with whom WOOD agrees.] In the other forms of lichen, depletion is not required.

In *psoriasis*, "in strong, healthy, plethoric young persons, when the eruption is of the guttated form, or affects only a small portion of the skin," venesection may precede other remedies. In some cases, the bleeding may be repeated, but not largely. [NELLIGAN, WOOD.]

In *pityriasis*, when there is considerable cutaneous inflammation, and the patient is vigorous, it may sometimes be proper to take blood from the arm; though this measure is not often required.

